# A case report: haemodynamic instability due to true dynamic left ventricular outflow tract obstruction and systolic anterior motion following resuscitation: reversal of haemodynamics on supportive veno-arterial extracorporeal membrane oxygenation

**DOI:** 10.1093/ehjcr/yty134

**Published:** 2018-11-27

**Authors:** Christina Stolzenburg Oxlund, Mikael Kjær Poulsen, Peter Blom Jensen, Karsten Tange Veien, Jacob Eifer Møller

**Affiliations:** 1 Department of Cardiology, University Hospital of Odense, Sdr. Boulevard 29, Odense C, Denmark; 2 Department of Vascular Intensive Care, University Hospital of Odense, Sdr. Boulevard 29, Odense C, Denmark

**Keywords:** Case report, Veno-arterial extracorporeal membrane oxygenation, Obstruction of the left ventricle outflow tract, Systolic anterior motion (SAM), Mitral regurgitation (MR)

## Abstract

**Background:**

Obstruction of the left ventricular outflow tract (LVOT) as seen in hypertrophic cardiomyopathy is a dynamic condition with a wide range of clinical presentations and symptoms.

**Case summary:**

We report the use of veno-arterial extracorporeal membrane oxygenation in a female patient who was resuscitated after out-of-hospital cardiac arrest. Soon after admission the patient developed critical haemodynamic compromise due to severe obstruction of the left ventricle outflow tract and systolic anterior motion (SAM) of the mitral valve. Veno-arterial extracorporeal membrane oxygenation restored haemodynamics and was weaned after 4 days without any haemodynamic compromise due to SAM. The patient was discharged from the intensive care unit at Day 13, and after 3 days at the coronary care unit, she was discharged to ambulatory follow-up with no sequelae.

**Discussion:**

Veno-arterial extracorporeal membrane oxygenation restored haemodynamic stability in this patient with dynamic severe LVOT obstruction following cardiac arrest.

**Learning points**

- Veno-arterial extracorporeal membrane oxygenation can restore haemodynamic stability in patients with transient severe left ventricular outflow tract obstruction following cardiac arrest and cardiogenic shock.
- Veno-arterial extracorporeal membrane oxygenation improves systemic circulation and oxygenation.

## Introduction

Obstruction of the left ventricular outflow tract (LVOTO) is most frequently seen as a consequence of septal myocardial hypertrophy in hypertrophic obstructive cardiomyopathy and in hypertensive heart disease. Systolic anterior motion (SAM) often accompanies LVOTO and is the result of high velocity blood flow in mid-systole, displacing the anterior mitral leaflet to the hypertrophied septum causing a dynamic obstruction throughout systole accompanied by mitral regurgitation (MR).^1^ Left ventricular outflow tract obstruction is associated with increased risk of sudden cardiac death, exercise intolerance, angina, and syncope. Left ventricular outflow tract obstruction is a dynamic condition and dependent on loading conditions of the left ventricle (LV) with worsening of the obstruction with reduced preload, decreased afterload, and increased contractility.^1^^,^^2^

## Timeline

**Table:** 

**Day 1**	Out of hospital Cardiac arrest intubation and respiratory support. Primary coronary intervention revealed chronic left anterior descending artery lesion. Echocardiogram with severe left ventricular outflow tract obstruction (LVOTO).
**Day 1–3**	Severe pulmonary congestion and low cardiac output with increasing lactate due to severe LVOTO and severe MR despite effort to increase afterload. Veno-arterial extracorporeal membrane oxygenation (VA-ECMO).
**Day 4**	Echocardiogram revealed cessation of MR and LVOTO. VA-ECMO removed.
**Day 5–12**	Stabilization at intensive care unit. Sedation weaning and patient extubation
**Day 13**	Implantable cardioverter-defibrillator implantation
**Day 16**	Discharged to cardiac rehabilitation

## Case presentation

A 74-year-old female with a history of hypertension and mild to moderate valvular aortic stenosis (AS) (mean gradient 20 mmHg) suffered cardiac arrest due to ventricular fibrillation (VF) at a restaurant. Bystander cardio pulmonary resuscitation was initiated immediately, and after two defibrillations she was cardioverted to atrial fibrillation. Return of spontaneous circulation was achieved after 10 min of CPR. The patient was intubated on site and transferred to the nearest primary coronary intervention centre. During transport she showed signs of awakening and was sedated with propofol. Adrenaline infusion was titrated to a mean arterial pressure (MAP) >70 mmHg.

The coronary angiogram showed severe proximal left anterior descending (LAD) artery stenosis. Complete revascularization was achieved after implantation of a single drug-eluting stent in the LAD artery. Troponin T values were only mildly elevated (257 ng/L) and the electrocardiogram without ST-segment elevation. The bedside transthoracic echocardiogram showed a hyperdynamic and hypertrophic left ventricle with ejection fraction of 50–60%. There were signs of LVOTO, SAM, and moderate MR, but no aortic regurgitation (AR).

The patient was transferred to the intensive care unit (ICU) for haemodynamic stabilization. Targeted temperature management was omitted due to haemodynamic instability and because the patient had been partly awake.

Within 6 h her haemodynamics deteriorated further with tachycardia 110–130 b.p.m., lactic acidosis (9.2 mmol/L), increasing demand for vasopressor therapy (norepinephrine: 0.7 μg/kg/min) to maintain MAP >60 mmHg and concurrent severe pulmonary oedema with a fall in peripheral oxygenation saturation to 60% despite 100% oxygenation supply and titration of positive end expiratory pressure settings of the mechanical ventilator. Efforts to increase pre- and afterload by volume and additional vasopressor therapy (vasopressin 1.0 U/kg/d) only worsened the degree of congestion.

**Figure 1:**
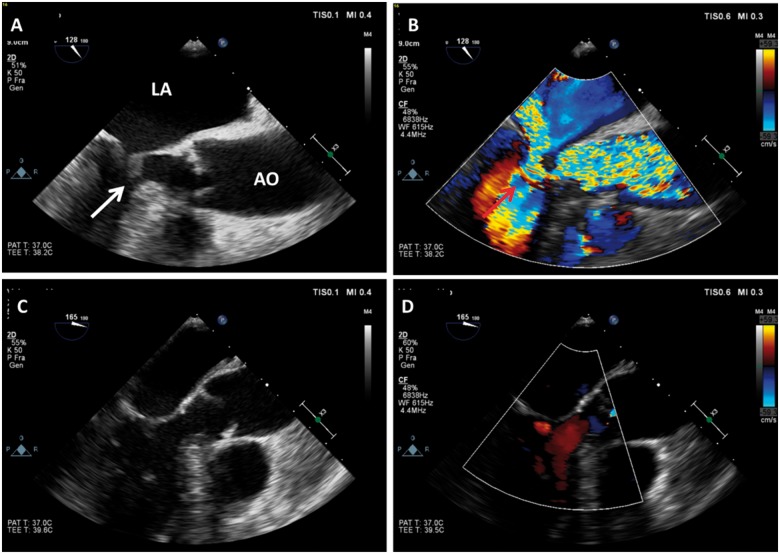
Transoesophageal echocardiographic systolic long-axis images. (*A* and *B*) Acquired before, and (*C* and *D*) Immediately after initiation of veno-arterial extracorporeal membrane oxygenation. (*A*) White arrow shows severe systolic anterior motion and (*B*) left ventricular outflow tract obstruction causing severe mitral regurgitation (red arrow). After initiation of veno-arterial extracorporeal membrane oxygenation systolic anterior motion, left ventricular outflow tract obstruction and mitral regurgitation disappeared.

Repeated transthoracic and transoesophageal echocardiograms revealed progression of LVOTO and SAM resulting in severe MR and pulmonary oedema (*Figure 1*; Supplementary material online, *Videos S1*–*S4*).

After a multidisciplinary team conference, it was decided to attempt to establish mechanical circulatory support with femoro-femoral veno-arterial extracorporeal membrane oxygenation (VA-ECMO) for oxygenation and restoration of tissue perfusion. Right femoral vein and artery was cannulated via Seldinger’s technique and VA-ECMO established using a Cardiohelp ECMO system. A 21 Fr venous catheter was placed in the right atrium guided by transoesophageal echocardiography for venous drainage. A 17 Fr arterial cannula was placed in the femoral artery and advanced for return of oxygenated blood. Veno-arterial extracorporeal membrane oxygenation blood flow was set at 4.0 L/min. This led to immediate improvement in haemodynamics and resolved SAM.

Lactate was normalized within 24 h and oxygenation improved. Demand for vasopressor therapy was minimized. On Day 4, haemodynamics had been restored. The echocardiogram revealed cessation of MR severity to mild and after VA-ECMO flow had been reduced to 1.5 L/min it was removed. After 5 days sedation weaning and extubation was achieved without complications despite intermittent use of low-dose norepinephrine to maintain MAP >70 mmHg.

A Day 13, the patient was discharged from the ICU to the coronary care unit. She was neurologically intact without echocardiographic signs of LVOTO or SAM. Overall, the patient was hospitalized for 16 days. She was discharged with beta-blocking agents after implantation of secondary prophylactic implantable cardioverter-defibrillator (ICD) to ambulatory cardiac rehabilitation. At 6 months of follow-up, the patient was in high spirit and fully asymptomatic. Transthoracic echocardiogram demonstrated normal ejection fraction, mild septal hypertrophy, and unchanged mild AS and discrete AR. No Doppler signs of MR.

Pressure gradient in LVOT at rest was not elevated. However during Valsalva manoeuvre the gradient in the LVOT increased to 42 mmHg, suggesting a latent LVOTO. The patient gave informed consent and all identifiable information has been removed.

## Discussion

Several reports have described that dynamic LVOTO may develop as complication in both ST-elevation myocardial infarction and Takotsubo cardiomyopathy in subjects with no previous history of LVOTO.^3^ In hypertrophic cardiomyopathy, the severity of LVOTO is associated with increased risk of sudden cardiac death. The risk increases with the severity of the obstruction and in symptomatic patients there is an indication for prophylactic ICD implantation.^4^ This patient had a history of mild to moderate AS and hypertensive heart disease, but no previous signs of hypertrophic cardiomyopathy with LVOTO.

Severe dynamic LVOTO may as in the present case mimic cardiogenic shock with inadequate tissue perfusion due to inadequate cardiac output and increased left ventricular filling pressures. This explains why our patient developed cardiogenic shock despite near to normal LVEF. Displacement of the anterior mitral leaflet in systole resulted not only in LVOTO worsening but also in coaptation failure of the anterior and posterior mitral leaflets with subsequent severe MR aggravating pulmonary oedema. Subvalvular LVOTO often occurs in combination with valvular AS. With AS, increased afterload tend to cause LV hypertrophy and subsequent of LVOTO. In hypertrophic obstructive cardiomyopathy, AS may appear by coincidence.^5^^,^^6^ The combined haemodynamic effects of valvular AS and LVOTO are low output syndrome and these effects might be synergistic. However, in this case, AS was only mild to moderate, suggesting haemodynamic instability was due to true LVOTO.

The post-cardiac arrest syndrome is characterized by disturbed pre- and afterload conditions, and in our patient systemic vascular resistance was low despite attempt to increase afterload with norepinephrine. Some case reports highlight that the use of inotropes like dobutamine attenuates dynamic obstruction causing a vicious circle.^7^

The Impella transvalvular device was considered unsuitable in the current situation due to SAM and hyperdynamic LV function and TandemHeart is not available at our centre thus VA-ECMO was chosen. Veno-arterial extracorporeal membrane oxygenation is a supportive treatment for cardiopulmonary resuscitation in patients with cardiogenic shock due to myocardial infarction, myocarditis, or pulmonary embolus with haemodynamic compromise and in some situations as bridge to decision for transplant or other definitive strategies.^8^ Veno-arterial extracorporeal membrane oxygenation establishes a right-to-left shunt by draining blood from the right atrium, blood is then oxygenated and warmed and returned to the arterial circulation in the iliac arteries.^9^ The purpose of the VA-ECMO is to improve systemic circulation and oxygenation, this at the cost of increased afterload that in the severely compromised heart may lead to inadequate myocardial unloading. Draining of venous blood from the right atrium using VA-ECMO reduce preload with potential of LVOTO aggravation and pulmonary congestion.^9^ However, LVOTO is not a contraindication to VA-ECMO as with severe AR. In the present case, VA-ECMO broke the vicious circle by restoring circulation and after weaning of mechanical circulatory support, and possibly due to increased afterload both the LVOTO and MR ceased.

To our knowledge, this is the first reported case of the use of VA-ECMO in severe dynamic LVOTO with haemodynamic compromise after cardiac arrest with primary VF without myocardial infraction. However, one case report describes the use of VA-ECMO in a patient with sustained cardiogenic shock due to dynamic LVOTO in Takotsubo cardiomyopathy (LVEF 25%). Veno-arterial extracorporeal membrane oxygenation completely reverted cardiogenic shock.^2^

## Conclusion

Veno-arterial extracorporeal membrane oxygenation can restore haemodynamic stability in patients with true dynamic LVOTO following cardiac arrest and cardiogenic shock. Thus, VA-ECMO can be used as potential *bridge to recovery* treatment in patients with cardiovascular collapse due to severe dynamic LVOTO.

**Slide sets:** A fully edited slide set detailing this case and suitable for local presentation is available online as Supplementary data.

**Consent:** The author/s confirm that written consent for submission and publication of this case report including image(s) and associated text has been obtained from the patient in line with COPE guidance.

**Conflict of interest:** none declared.

## Supplementary Material

Supplementary Data
