# Supplementary material for: A case report: haemodynamic instability due to true dynamic left ventricular outflow tract obstruction and systolic anterior motion following resuscitation: reversal of haemodynamics on supportive veno-arterial extracorporeal membrane oxygenation
Source: Eur Heart J Case Rep. 2018 Nov 27;2(4):yty134. doi: 10.1093/ehjcr/yty134 (PMC6426033; doi:10.1093/ehjcr/yty134)
Supplement: Supplementary Data [file yty134_supp.pptx]

## Slide 1
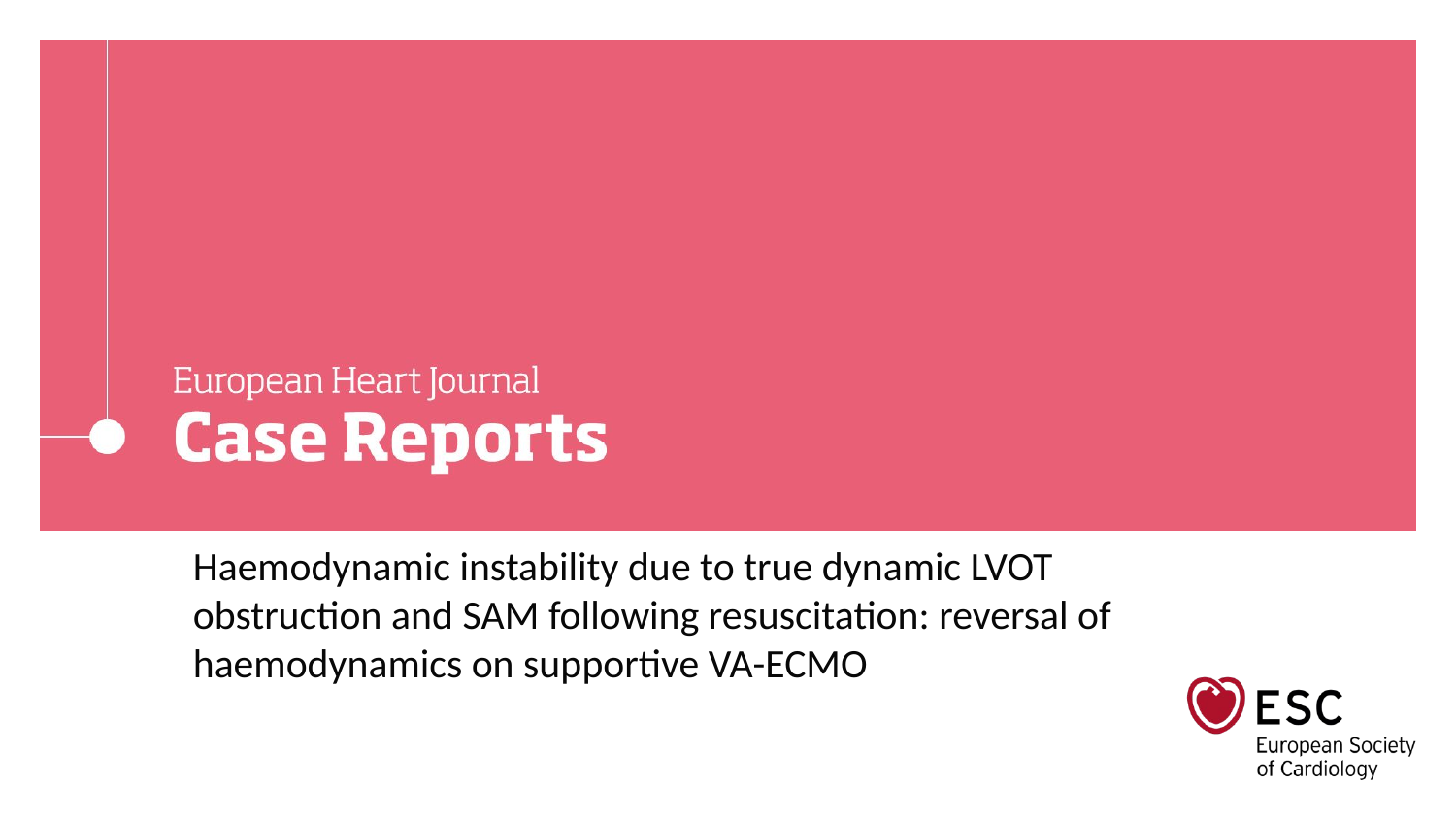

# Haemodynamic instability due to true dynamic LVOT obstruction and SAM following resuscitation: reversal of haemodynamics on supportive VA-ECMO

## Slide 2
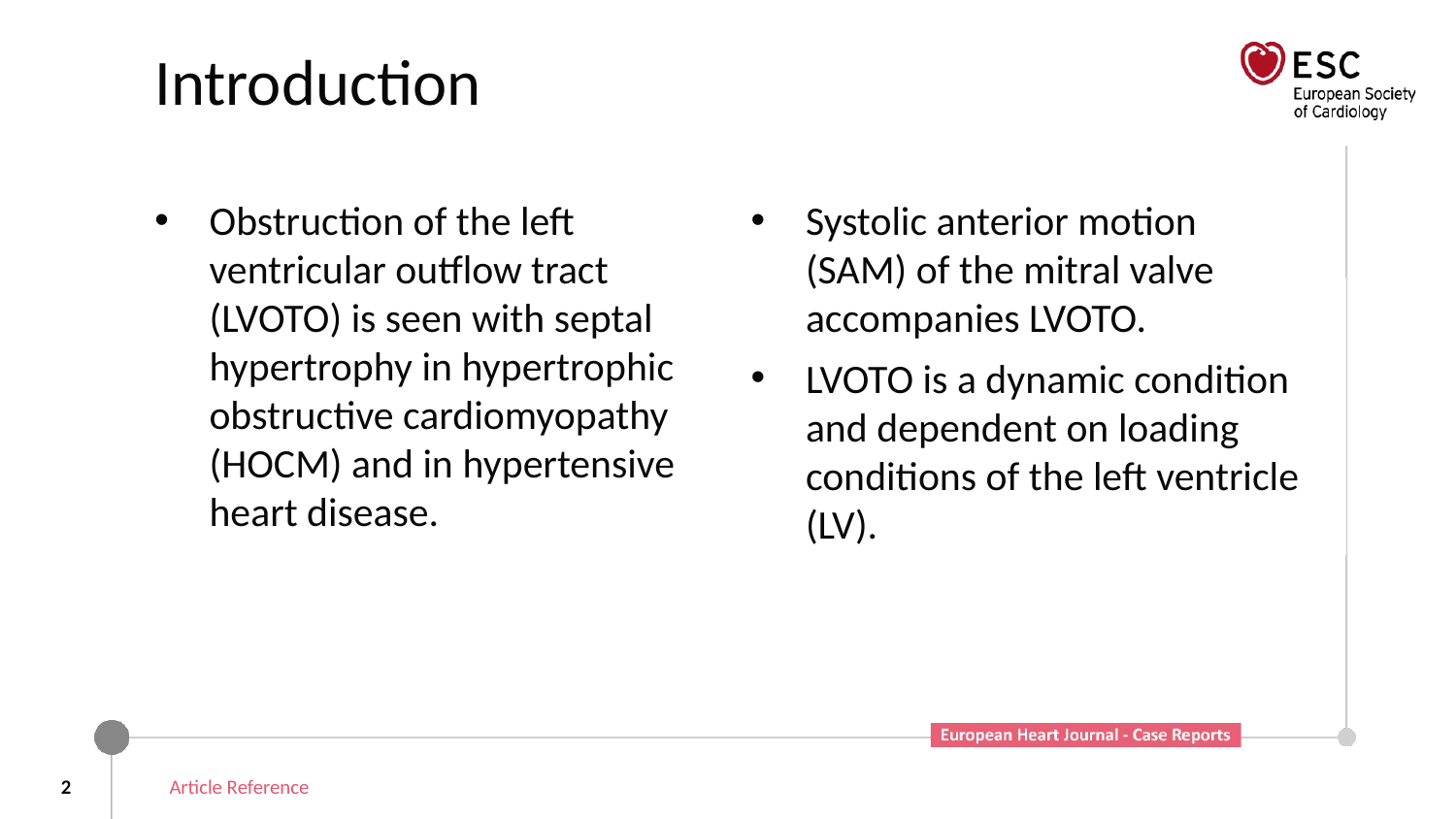

# Introduction
Obstruction of the left ventricular outflow tract (LVOTO) is seen with septal hypertrophy in hypertrophic obstructive cardiomyopathy (HOCM) and in hypertensive heart disease.
Systolic anterior motion (SAM) of the mitral valve accompanies LVOTO.
LVOTO is a dynamic condition and dependent on loading conditions of the left ventricle (LV).
2
Article Reference

## Slide 3
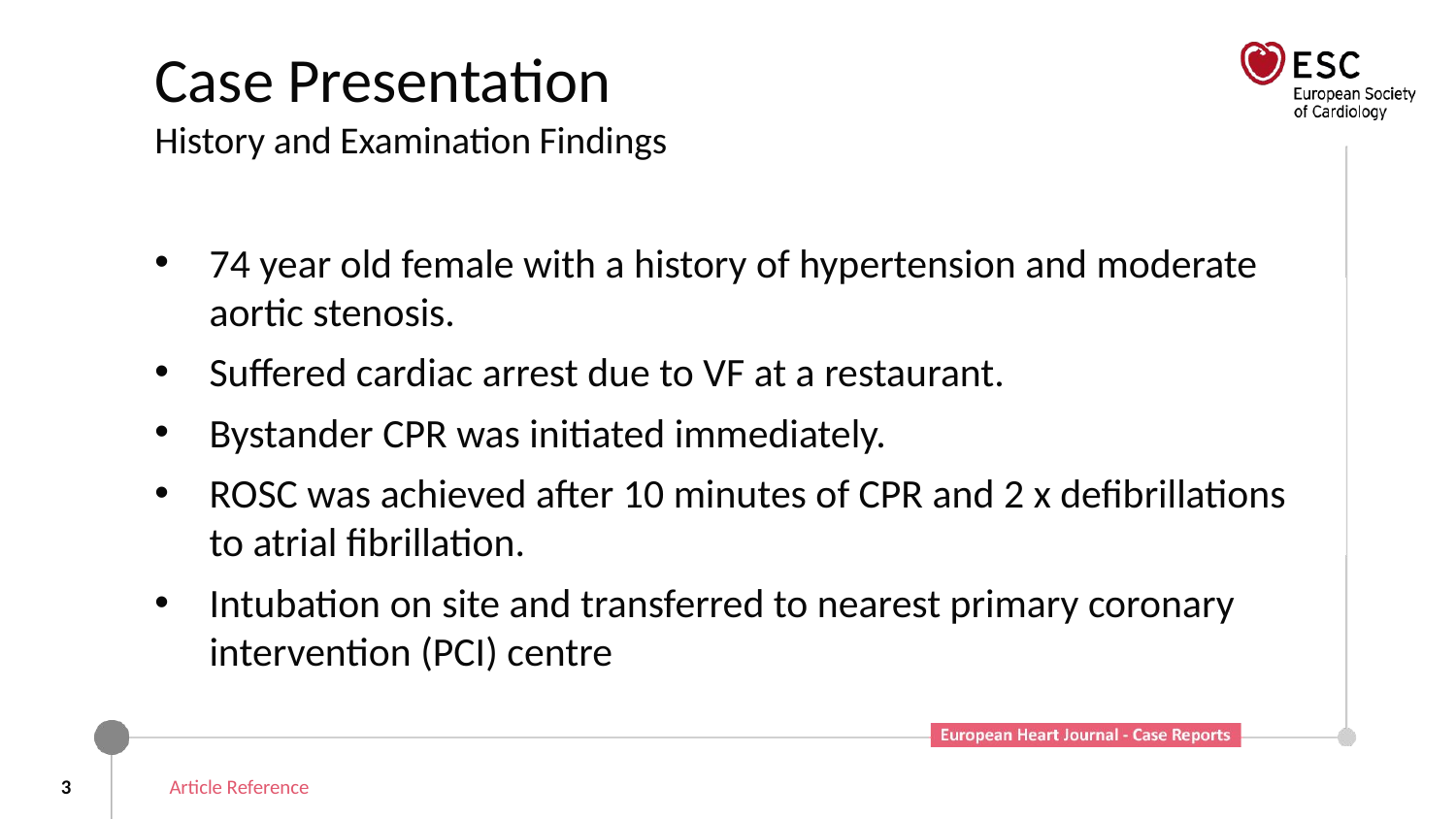

# Case Presentation History and Examination Findings
74 year old female with a history of hypertension and moderate aortic stenosis.
Suffered cardiac arrest due to VF at a restaurant.
Bystander CPR was initiated immediately.
ROSC was achieved after 10 minutes of CPR and 2 x defibrillations to atrial fibrillation.
Intubation on site and transferred to nearest primary coronary intervention (PCI) centre
3
Article Reference

## Slide 4
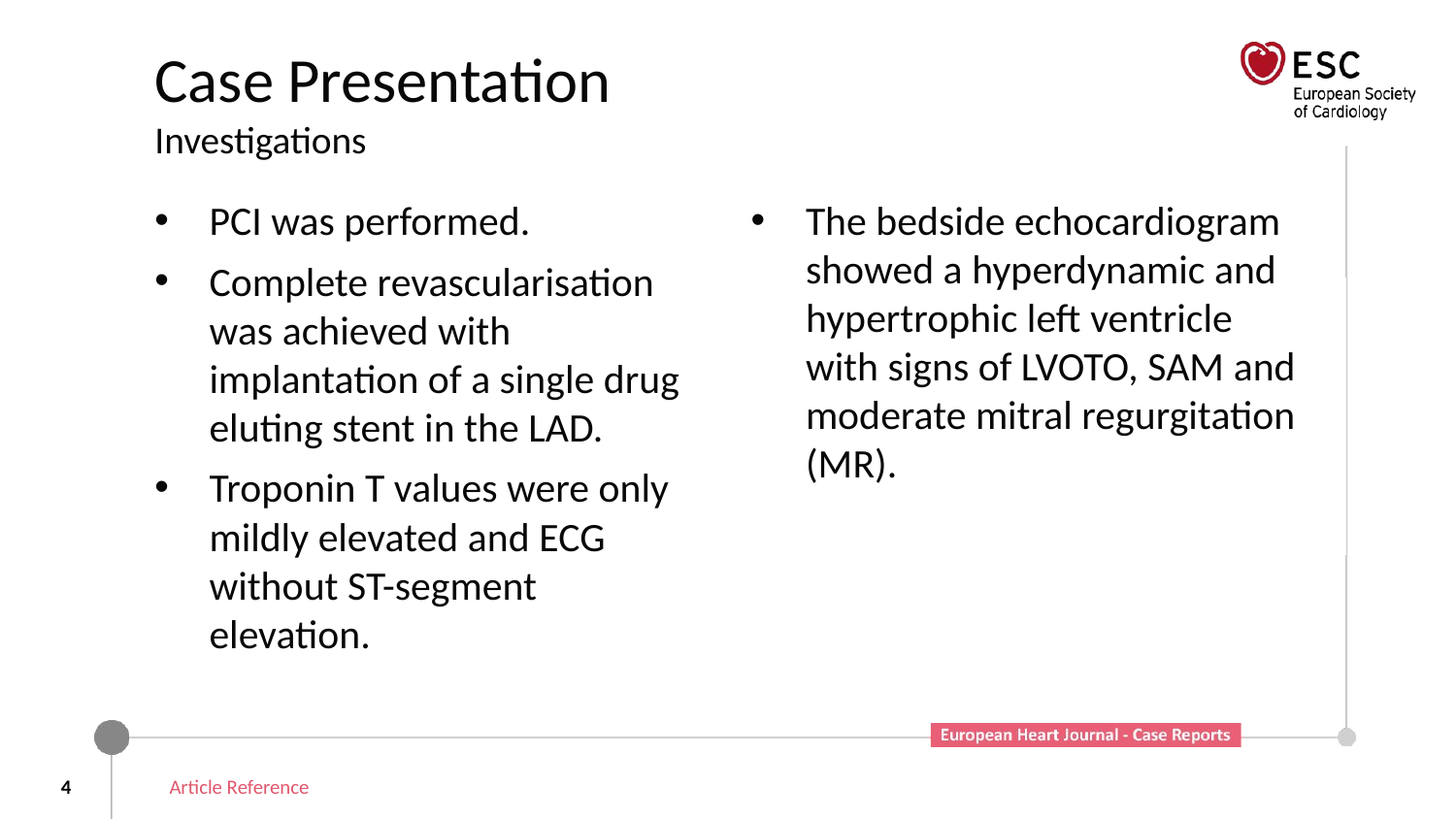

# Case Presentation Investigations
PCI was performed.
Complete revascularisation was achieved with implantation of a single drug eluting stent in the LAD.
Troponin T values were only mildly elevated and ECG without ST-segment elevation.
The bedside echocardiogram showed a hyperdynamic and hypertrophic left ventricle with signs of LVOTO, SAM and moderate mitral regurgitation (MR).
4
Article Reference

## Slide 5
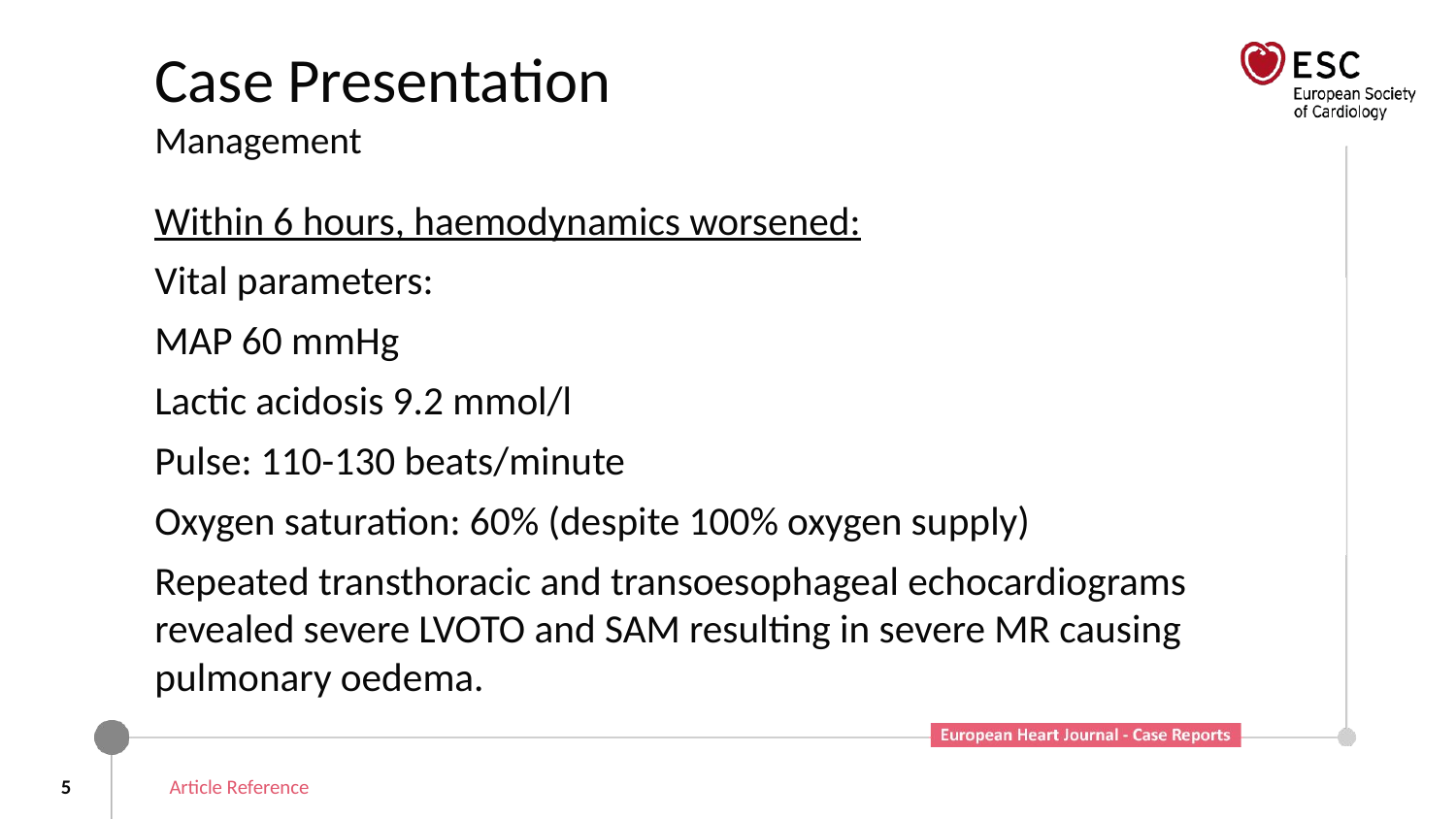

# Case Presentation Management
Within 6 hours, haemodynamics worsened:
Vital parameters:
MAP 60 mmHg
Lactic acidosis 9.2 mmol/l
Pulse: 110-130 beats/minute
Oxygen saturation: 60% (despite 100% oxygen supply)
Repeated transthoracic and transoesophageal echocardiograms revealed severe LVOTO and SAM resulting in severe MR causing pulmonary oedema.
5
Article Reference

## Slide 6
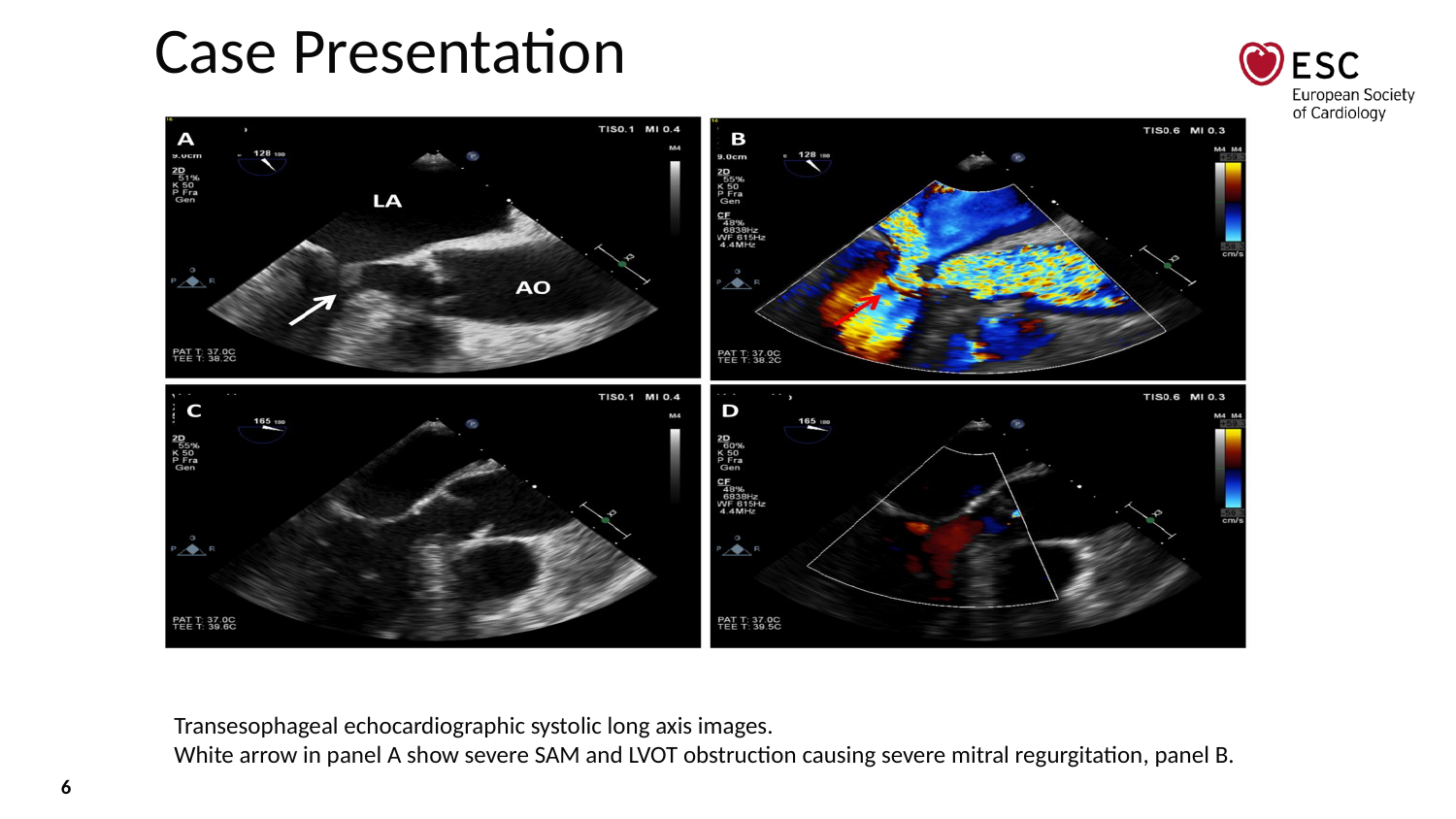

# Case Presentation
Transesophageal echocardiographic systolic long axis images.
White arrow in panel A show severe SAM and LVOT obstruction causing severe mitral regurgitation, panel B.
6

## Slide 7
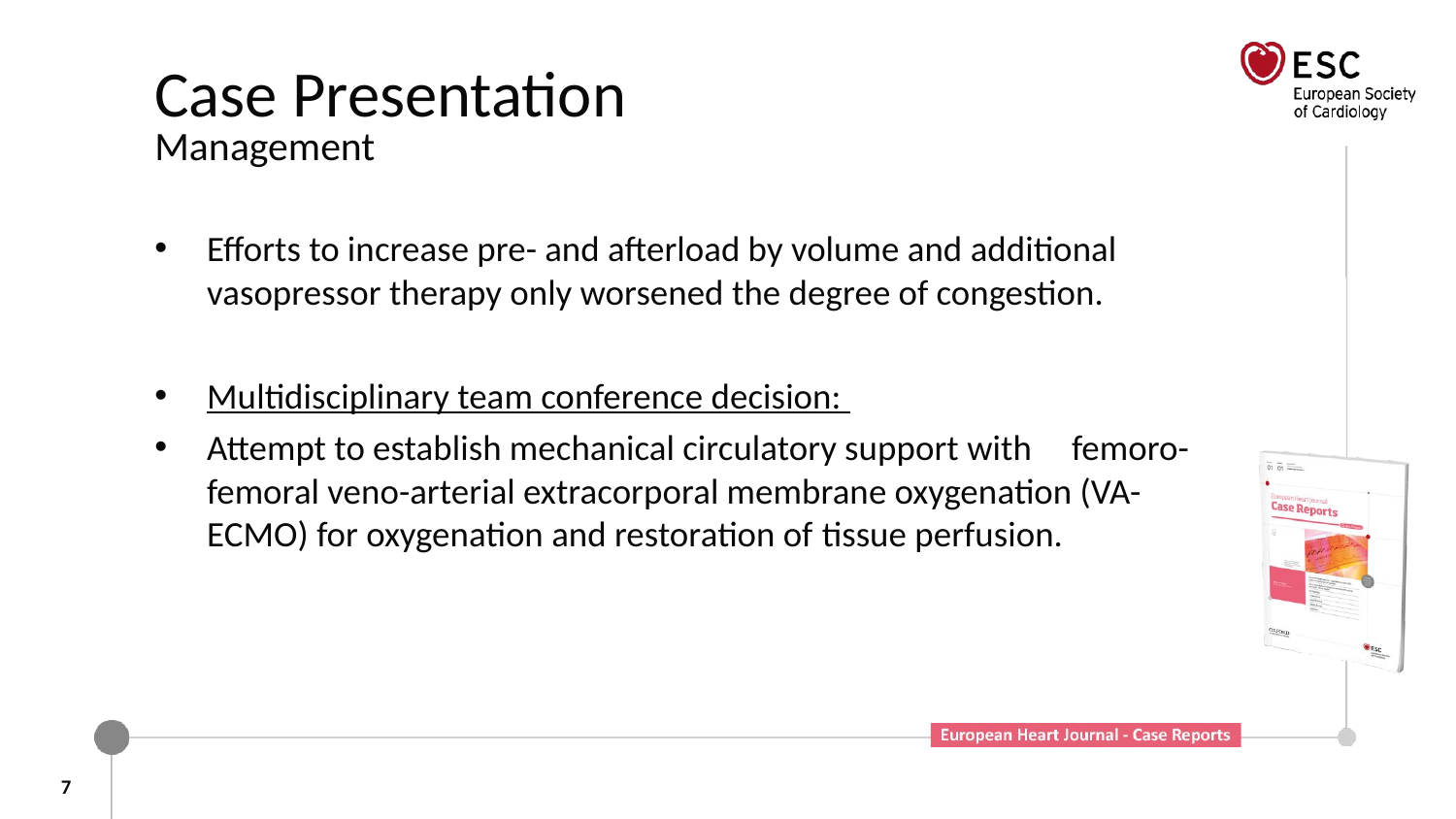

# Case Presentation Management
Efforts to increase pre- and afterload by volume and additional vasopressor therapy only worsened the degree of congestion.
Multidisciplinary team conference decision:
Attempt to establish mechanical circulatory support with femoro-femoral veno-arterial extracorporal membrane oxygenation (VA-ECMO) for oxygenation and restoration of tissue perfusion.
7

## Slide 8
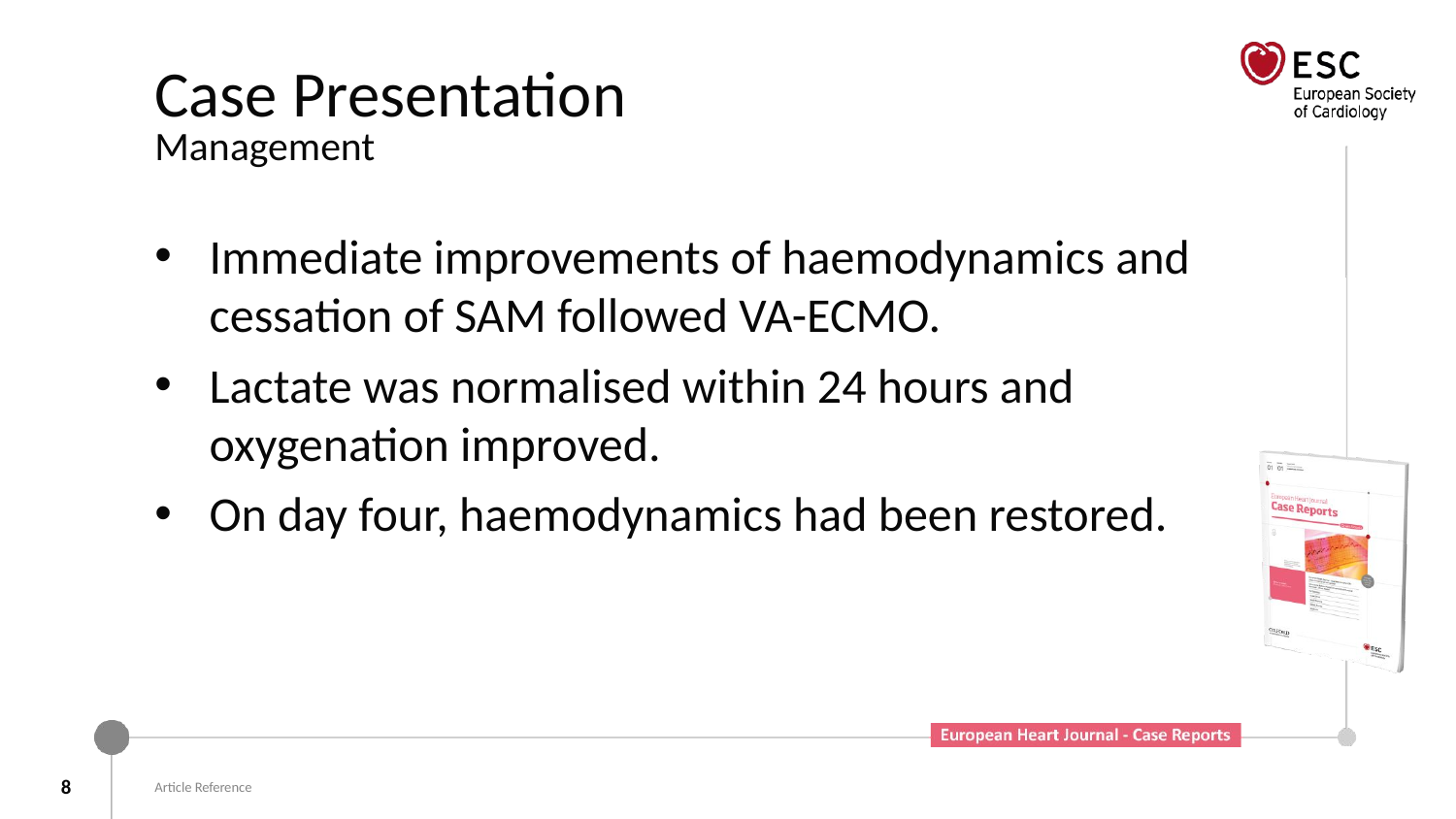

# Case Presentation Management
Immediate improvements of haemodynamics and cessation of SAM followed VA-ECMO.
Lactate was normalised within 24 hours and oxygenation improved.
On day four, haemodynamics had been restored.
8
Article Reference

## Slide 9
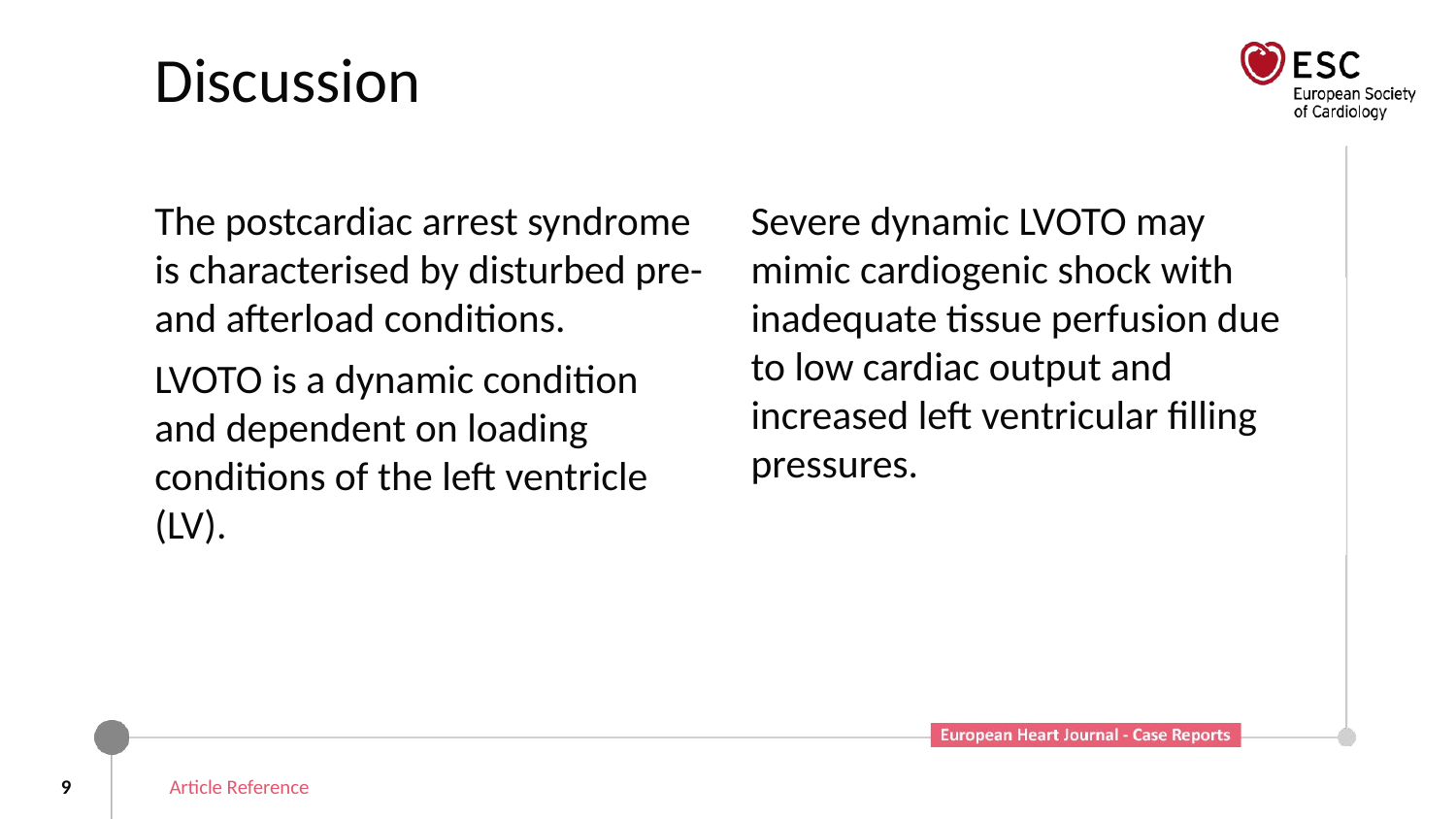

# Discussion
The postcardiac arrest syndrome is characterised by disturbed pre- and afterload conditions.
LVOTO is a dynamic condition and dependent on loading conditions of the left ventricle (LV).
Severe dynamic LVOTO may mimic cardiogenic shock with inadequate tissue perfusion due to low cardiac output and increased left ventricular filling pressures.
9
Article Reference

## Slide 10
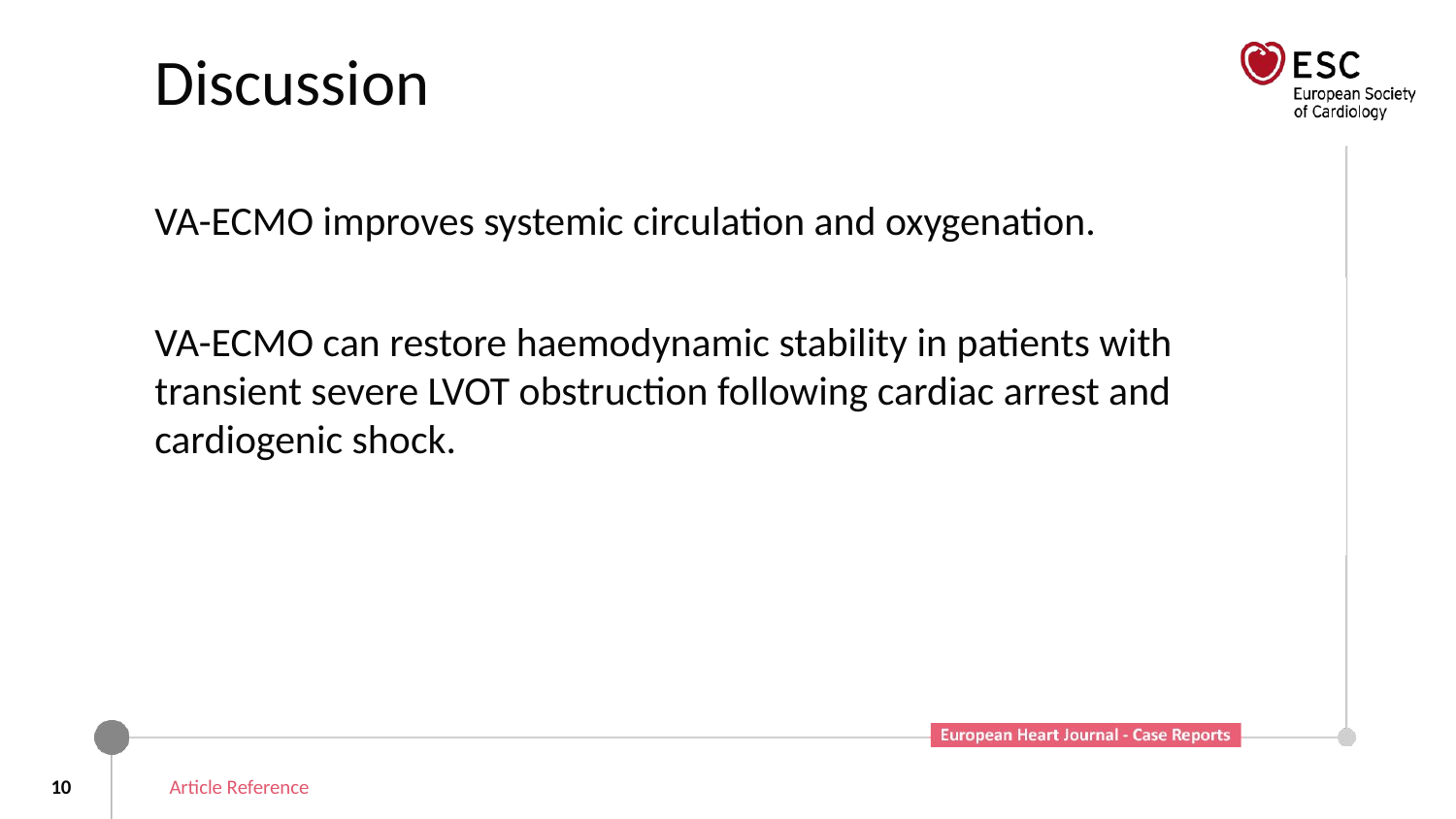

# Discussion
VA-ECMO improves systemic circulation and oxygenation.
VA-ECMO can restore haemodynamic stability in patients with transient severe LVOT obstruction following cardiac arrest and cardiogenic shock.
10
Article Reference

## Slide 11
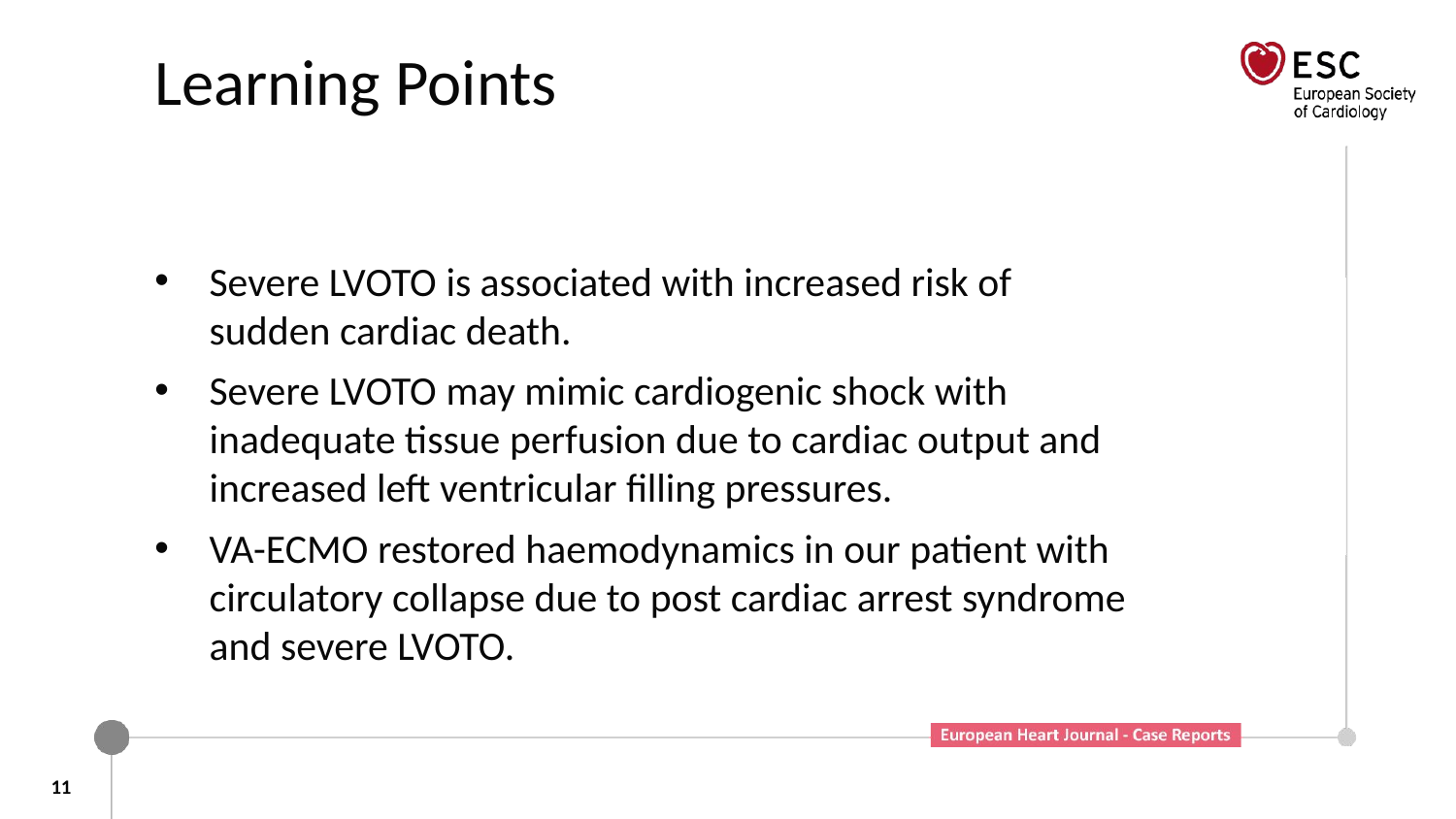

# Learning Points
Severe LVOTO is associated with increased risk of sudden cardiac death.
Severe LVOTO may mimic cardiogenic shock with inadequate tissue perfusion due to cardiac output and increased left ventricular filling pressures.
VA-ECMO restored haemodynamics in our patient with circulatory collapse due to post cardiac arrest syndrome and severe LVOTO.
11

## Slide 12
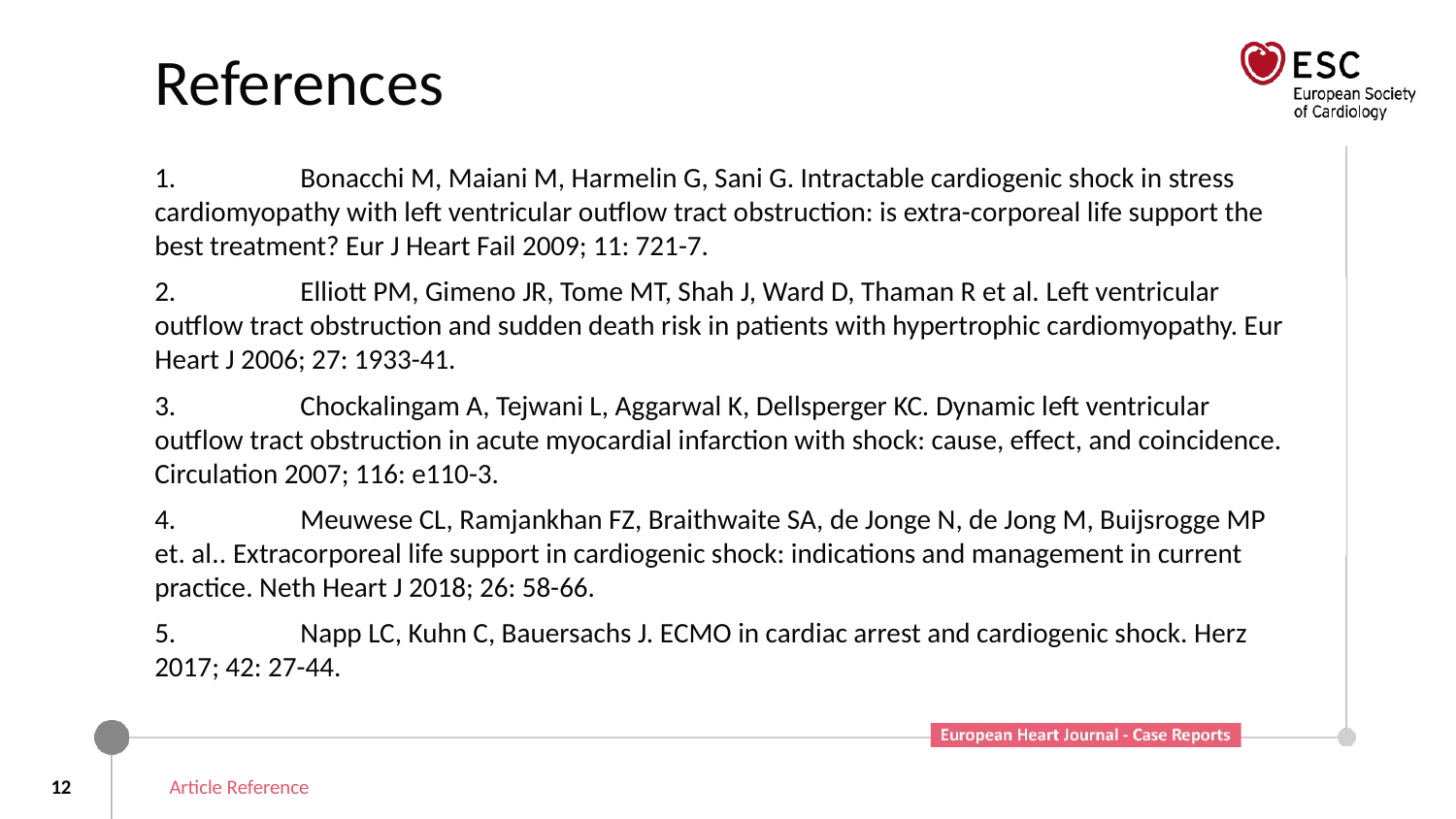

# References
1.	Bonacchi M, Maiani M, Harmelin G, Sani G. Intractable cardiogenic shock in stress cardiomyopathy with left ventricular outflow tract obstruction: is extra-corporeal life support the best treatment? Eur J Heart Fail 2009; 11: 721-7.
2.	Elliott PM, Gimeno JR, Tome MT, Shah J, Ward D, Thaman R et al. Left ventricular outflow tract obstruction and sudden death risk in patients with hypertrophic cardiomyopathy. Eur Heart J 2006; 27: 1933-41.
3.	Chockalingam A, Tejwani L, Aggarwal K, Dellsperger KC. Dynamic left ventricular outflow tract obstruction in acute myocardial infarction with shock: cause, effect, and coincidence. Circulation 2007; 116: e110-3.
4.	Meuwese CL, Ramjankhan FZ, Braithwaite SA, de Jonge N, de Jong M, Buijsrogge MP et. al.. Extracorporeal life support in cardiogenic shock: indications and management in current practice. Neth Heart J 2018; 26: 58-66.
5.	Napp LC, Kuhn C, Bauersachs J. ECMO in cardiac arrest and cardiogenic shock. Herz 2017; 42: 27-44.
12
Article Reference
